# Reproducibility of repeated breathhold and impact of breathhold failure in whole breast and regional nodal irradiation in prone crawl position

**DOI:** 10.1038/s41598-022-05957-7

**Published:** 2022-02-03

**Authors:** Pieter Deseyne, Bruno Speleers, Leen Paelinck, Werner De Gersem, Wilfried De Neve, Max Schoepen, Annick Van Greveling, Hans Van Hulle, Vincent Vakaet, Giselle Post, Chris Monten, Herman Depypere, Liv Veldeman

**Affiliations:** 1 Department of Radiation Oncology, Ghent University Hospital, Corneel Heymanslaan 10, 9000 Ghent, Belgium; 2 Department of Human Structure and Repair, Faculty of Medicine and Health Sciences, Ghent University, Ghent, Belgium; 3 Department of Industrial Systems Engineering and Product Design, Faculty of Engineering and Architecture, Ghent University, Ghent, Belgium; 4 Department of Obstetrics and Gynaecology, Ghent University Hospital, Ghent, Belgium

**Keywords:** Breast cancer, Breast cancer, Breast cancer

## Abstract

In whole breast and regional nodal irradiation (WB + RNI), breathhold increases organ at risk (OAR) sparing. WB + RNI is usually performed in supine position, because positioning materials obstruct beam paths in prone position. Recent advancements allow prone WB + RNI (pWB + RNI) with increased sparing of OARs compared to supine WB + RNI. We evaluate positional and dosimetrical impact of repeated breathhold (RBH) and failure to breathhold (FTBH) in pWB + RNI. Twenty left-sided breast cancer patients were scanned twice in breathhold (baseline and RBH) and once free breathing (i.e. FTBH). Positional impact was evaluated using overlap index (OI) and Dice similarity coefficient (DSC). Dosimetrical impact was assessed by beam transposition from the baseline plan. Mean OI and DSC ranges were 0.01–0.98 and 0.01–0.92 for FTBH, and 0.73–1 and 0.69–1 for RBH. Dosimetric impact of RBH was negligible. FTBH significantly decreased minimal dose to CTV WBI, level II and the internal mammary nodes, with adequate mean doses. FTBH significantly increased heart, LAD, left lung and esophagus dose. OI and DSC for RBH and FTBH show reproducible large ROI positions. Small ROIs show poor overlap. FTBH maintained adequate target coverage but increased heart, LAD, ipsilateral lung and esophagus dose. RBH is a robust technique in pWB + RNI. (Clinicaltrials.gov: NCT05179161, registered 05/01/2022).

## Introduction

Therapeutic evolution has led to an increase in long-term survival for breast cancer patients. As survival increases, so does the impact of treatment-related side effects. The effects of radiotherapy on the occurrence of major cardiac events have been described^1^. For this reason, several techniques have been proposed to limit the dose to the heart. The most important are breathhold^2,3^ and prone positioning^4,5^.

Breathhold can be used in whole breast and regional nodal irradiation (WB + RNI) to improve sparing of normal tissues^6,7^. In the past, there have been several reports of patients treated in prone position for WB + RNI, but in these cases, the breast board on which the patient is positioned, obstructs beam access to the regional nodal areas, leading to either a bolus effect or necessitating beam entry trough healthy tissues. Therefore, the prone position is almost exclusively used in WBI, and not in WB + RNI. However, recent trials have shown that WB + RNI in prone position is possible and might come with the same benefits of prone positioning observed in WBI^8–12^.

It has been shown that it is feasible to combine breathholding and prone positioning for WBI with high reproducibility of the breast position^3,13,14^. A recent meta-analysis by Lai et al. even hinted that the combined use of prone positioning and breathholding seemed the most promising to decrease cardiopulmonary exposure, rather than breathholding or prone positioning on their own^15^. In our previous research, we showed a benefit of prone positioning over supine positioning in WB + RNI^9,12^, and in another paper that the addition of breathhold for WB + RNI in prone position was more beneficial than prone positioning alone in photon beam treatment^16^. But contrary to the breast itself in prone position^14^, nodal target volume locations may vary significantly between shallow breathing and breathhold, requiring strict breathhold monitoring to avoid missing the target. These observations have been reported for supine position^17,18^, but such data are non-existent for prone positioning.

This trial investigates the reproducibility of the previously observed beneficial combination of prone positioning and breathhold in WB + RNI, and the impact of the observed changes on dose coverage. The variation in position of organs at risk (OARs) and target volumes (TVs) between breathhold and free breathing in prone position is evaluated, to estimate the impact of situations when patients do not succeed in maintaining breathhold during treatment.

## Materials and methods

### Study design

This study investigates the feasibility and intra-fraction reproducibility of the breathhold technique in prone position for patients requiring WB + RNI. We included 20 left-sided breast cancer patients treated with breast conserving surgery and who had no nodal involvement. Median patient age was 54 (range 37–76). All patients were referred to our center for WBI but we used their simulation imaging to plan the WBI + RNI treatments investigated in this trial. Main inclusion criteria were adult female breast cancer patients without metastases requiring WBI only, after multidisciplinary tumour board discussion consensus. Main exclusion criteria were mastectomy, requirement of partial breast irradiation or regional nodal irradiation, and inability to be treated in prone position. No adverse events related to the patient positioning occurred. For the purpose of this trial, all patients were considered to have had breast conserving surgery and positive axillary lymph node dissection, i.e. the nodal positive patients in stages IIA through IIIC. In practice, all patients received WBI only. In addition to a free breathing computed tomography (CT) scan, patients underwent 2 voluntary deep inspiration breathhold (BH) scans at simulation. The second BH scan was performed to evaluate reproducibility of the procedure, and had no therapeutic implications. The trial was approved by the Ghent University Hospital Ethics Board (reference number: EC-UZ-2016/0351, Belgian Registration Number: B670201628048). All patients in our research voluntarily joined the study and informed consent was obtained from all participants before inclusion. The research was performed in accordance with relevant guidelines and regulations.

### Prone crawl position

Patients were positioned in prone position on the crawl couch, which was specifically designed for WB + RNI. The device is described elsewhere and yields lower doses to OARs while maintaining target coverage when compared to supine WB + RNI^9,12,19^.

### Simulation and breathhold

All patients underwent CT simulation with a unilateral bra [Tricolast, Deinze, Belgium] that retracts the contralateral breast from the TVs. We used a slice thickness of 5 mm for image acquisition. Each patient underwent a free breathing CT scan and a BH scan, as well as an additional low dose repeated breathhold (RBH) scan. For both BH and the RBH scan, the breathholding manoeuvre was performed according to procedures previously published elsewhere^20^. The RBH scan was performed to investigate the repeatability of the BH manoeuvre, because patients have to repeat the manoeuvre multiple times during treatments due to beam-on times being too long to complete in a single breathholding manoeuvre. Patient position nor scan range were altered between each scan, assuring that the DICOM coordinate system, indicated by the frame of reference unique identifier of the different scans, remained identical. The RBH manoeuvre was monitored using 2 Respisens magnetic sensors (Nomics, Angleur, Belgium) placed on the breast couch and thoracic wall^20^. Patient position and sensor placement is illustrated in a figure in the publication by Speleers et al.^16^ No IV-contrast was administered. This provided us with a set of 3 scans of distinct clinical situations for each patient, namely BH, RBH and free breathing, which for further purposes of this publication we will call failure to breathhold (FTBH). We will further refer to these names when describing these clinical situations, where we consider BH to be the index situation and RBH and FTBH the comparative situations.

### Treatment design and prescribed dose

The prescription dose was 40.05 Gy in 15 fractions to the whole breast, axillary levels II-III-IV and internal mammary nodes. Level I was excluded from the target volumes because this level is cleared during axillary lymph node dissection.

### Delineation

An extrapolation of the accepted guidelines for delineation of the breast and lymphatic regions from the contouring guidelines as proposed by the ESTRO and PROCAB groups^21–24^ was used as there are no generally accepted guidelines for delineation in prone position. The axillary levels I-II-III (LNN I, LNN II and LNN III), the supraclavicular fossa (LNN IV) and internal mammary nodes (LNN MI) were delineated separately, resulting in 5 separate nodal regions. The heart and left anterior descending coronary artery (LAD) were delineated in accordance with guidelines proposed by Feng et al.^25^. The contralateral breast was delineated up to the skin. Where OARs were not visible on certain CT-slices, interpolation was used. Because all images were acquired within the same session without repositioning, rigid co-registration by DICOM coordinates was performed. This way, the co-registration resembles what happens during a treatment fraction. As this is part of our regular patient flow for WBI patients, delineations were first performed on the FTBH scan, and subsequently were copied to the BH scan and adapted where necessary. This process was repeated between the BH and the RBH scan. All registrations and delineations were performed using RayStation 9 (RaySearch Laboratories, Stockholm, Sweden).

### Overlap indicators

After contouring the TVs and OARs, we performed a paired comparison of the total volumes of the breast, individual nodal regions, heart, LAD, and contralateral breast between BH and RBH scans, and between FTBH and BH scans. We assessed the spatial overlap between contoured volumes on different scans using the Dice similarity coefficient (DSC)^26^ and the overlap index (OI)^14^. Both have been used to describe reproducibility and accuracy of delineation.

The DSC is defined as follows:

$$DSC= \frac{2 \times (A \cap B)}{A+B}$$

whereas the OI is defined as:

$$OI =\frac{A\cap B}{A}$$

In both formulas, A is the volume of a contoured region on one scan, and B is the volume of the same contoured region on another scan. Data were obtained through the scripting module provided with RayStation 9 after co-registration and contour verification.

These numerical values are a surrogate of the clinical relevance of reproducibility. Therefore, we also investigated dosimetry on these scans to assess the impact of the observed overlap indicators.

### Dosimetric estimation of clinical relevance of intra-fraction motion

Because we investigate BH robustness, a treatment plan was made for the BH scan for each patient using a previously described technique^9,12,16^. In short, treatment is designed using multiple short non-coplaner VMAT arcs. Figure 1 from Speleers et al.^16^ shows the beam angles used. The linear accelerator for which the treatment was planned is an Elekta Synergy (Elekta, Stockholm, Sweden) The treatment beams for this initial plan were transposed by copying the machine instruction file to the FTBH scan. The dose was then recalculated without re-optimization on the FTBH scan to assess the effect of FTBH. The same was done on the RBH scan to assess the reproducibility of RBH. A separately measured electron-density table was used for dose calculation on the low-dose CT.

### Data analysis

Data was analysed using R version 4.1.1, using two-sided paired significance testing with an α-level of 0.05.

## Results

### Absolute volumes

Paired T-tests showed a significant absolute volume difference for the contralateral breast, heart, lungs and level II (P < 0.05) between BH and FTBH scans. Only left and right lung volume was significantly different between BH and RBH scans. Table 1 shows the mean volumes and standard deviation for each of the delineated regions of interest (ROIs) on the different scans.

**Table 1:** Mean volume ± standard deviation (sd) in milliliter for each delineated volume on all scans.

	BH	RBH			FTBH		
	Volume (ml) ± sd	Volume (ml) ± sd	T-statistic	P-value	Volume (ml) ± sd	T-statistic	P-value
CTV WBI	484.59 ± 347.02	456.74 ± 298.29	0.841	0.411	455.62 ± 298.56	0.862	0.399
LNN I	119.23 ± 57.08	119.68 ± 57.34	− 1.321	0.202	117.96 ± 56.88	0.456	0.654
LNN II	16.22 ± 7.25	15.90 ± 7.36	1.843	0.081	18.02 ± 7.74 *	− 3.834	0.001
LNN III	14.02 ± 4.64	14.05 ± 4.62	− 0.278	0.784	13.73 ± 4.19	0.372	0.714
LNN IV	12.35 ± 3.20	12.41 ± 3.18	− 0.495	0.526	13.11 ± 2.63	− 1.434	0.168
LNN MI	7.62 ± 3.00	7.59 ± 2.65	0.251	0.805	8.42 ± 2.32	− 1.486	0.154
Heart	604.68 ± 73.64	602.29 ± 76.84	0.789	0.440	648.03 ± 100.18	− 4.799	< 0.001
LAD	3.98 ± 1.77	4.02 ± 1.66	− 0.553	0.587	3.80 ± 1.72	0.649	0.524
Left Lung	2313.95 ± 473.40	2275.19 ± 458.07	2.198	0.041	1562.78 ± 365.86	13.114	< 0.001
Right Lung	2634.98 ± 458.17	2516.19 ± 472.28	4.177	0.001	1875.46 ± 394.72	13.069	< 0.001
Esophagus	32.16 ± 14.55	31.11 ± 12.34	1.130	0.273	29.94 ± 12.20	1.344	0.195
Thyroid	17.28 ± 15.20	16.69 ± 15.59	0.795	0.437	16.39 ± 14.67	1.165	0.258
Contralat breast	606.14 ± 359.42	606.286 ± 359.73	− 0.638	0.531	618.80 ± 369.03	− 3.073	0.006

Tests report comparisons of either FTBH or RBH to BH.

*FTBH* scan in failure to breathhold setting, *BH* initial voluntary deep inspiration breathhold scan, *RBH* low dose repeated breathhold scan, *CTV WBI* clinical target volume of the treated breast, *LAD* left anterior descendant coronary artery, *Contralat.* Contralateral, *LNN I through IV* lymph node levels I–IV, *LNN MI* internal mammary nodes.

### Overlap measurements

**Figure 1:**
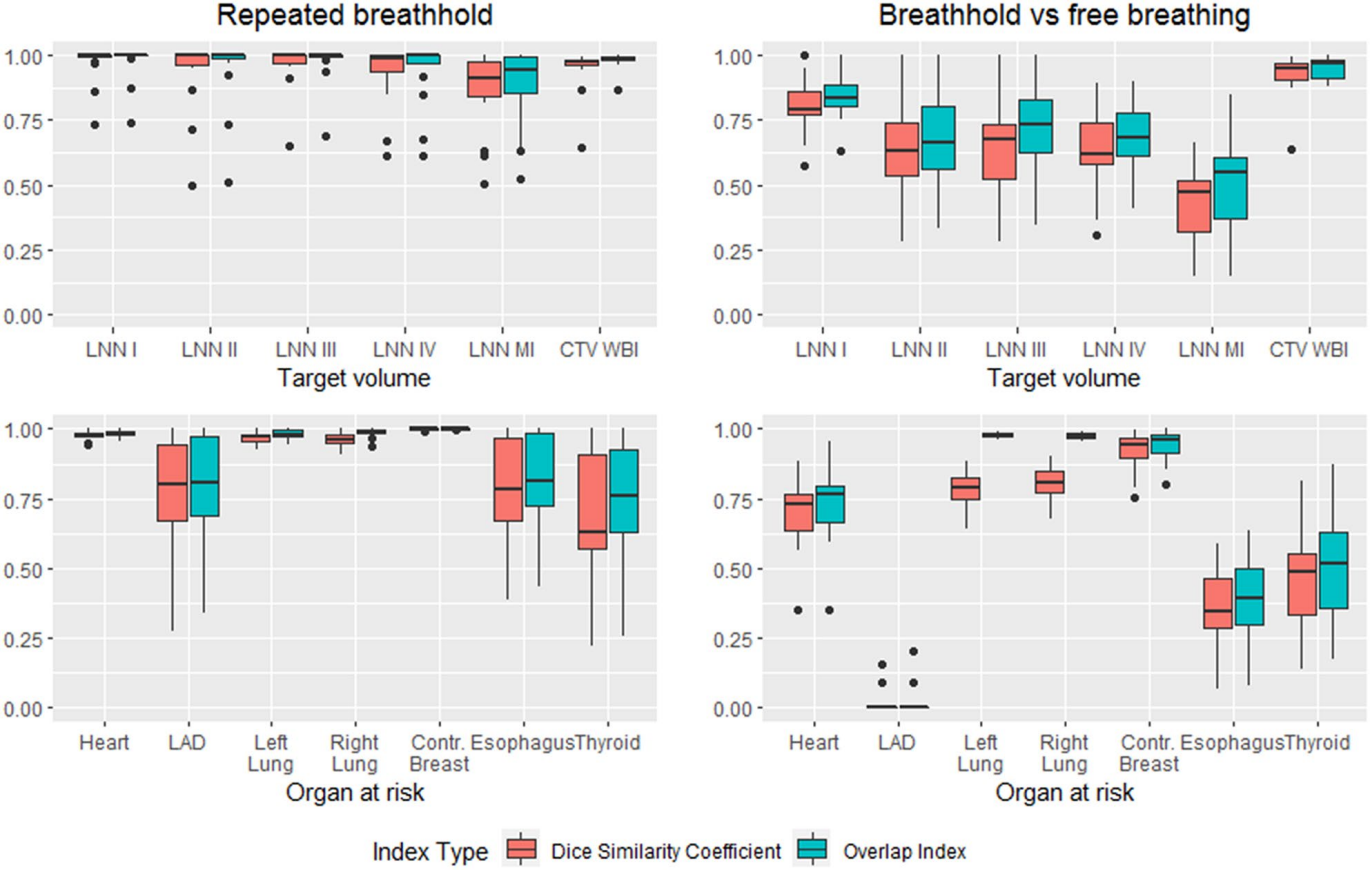
Boxplot of the overlap measurements for targets and organs at risk for the repeated breathhold and failure to breathhold setting. The top row shows target volumes and the bottom row shows OARs, while the first column shows the situation for repeated breathhold and the second column shows the situation for failure to breathhold. Whiskers show 1.5 * interquartile range, outliers plotted as dots. Overlap index is consistently higher than Dice similarity coefficient for the same ROIs in the same breathing phase setting. Small volumes have poorer overlap measurements. This is especially visible in failure to breathhold setting.

Figure 1 shows a boxplot of OI and DSC for the different ROIs in the different breathing settings. Regarding OARs, LAD, esophagus and thyroid showed more variability in OI and DSC than the other OARs. For TVs, OI and DSC varied more prominently. All ROIs with smaller volumes show higher overlap discrepancies. OI and DSC show a high correlation (Pearson’s r = 0.98, R^2^ = 0.95).

### Beam transposition

**Figure 2:**
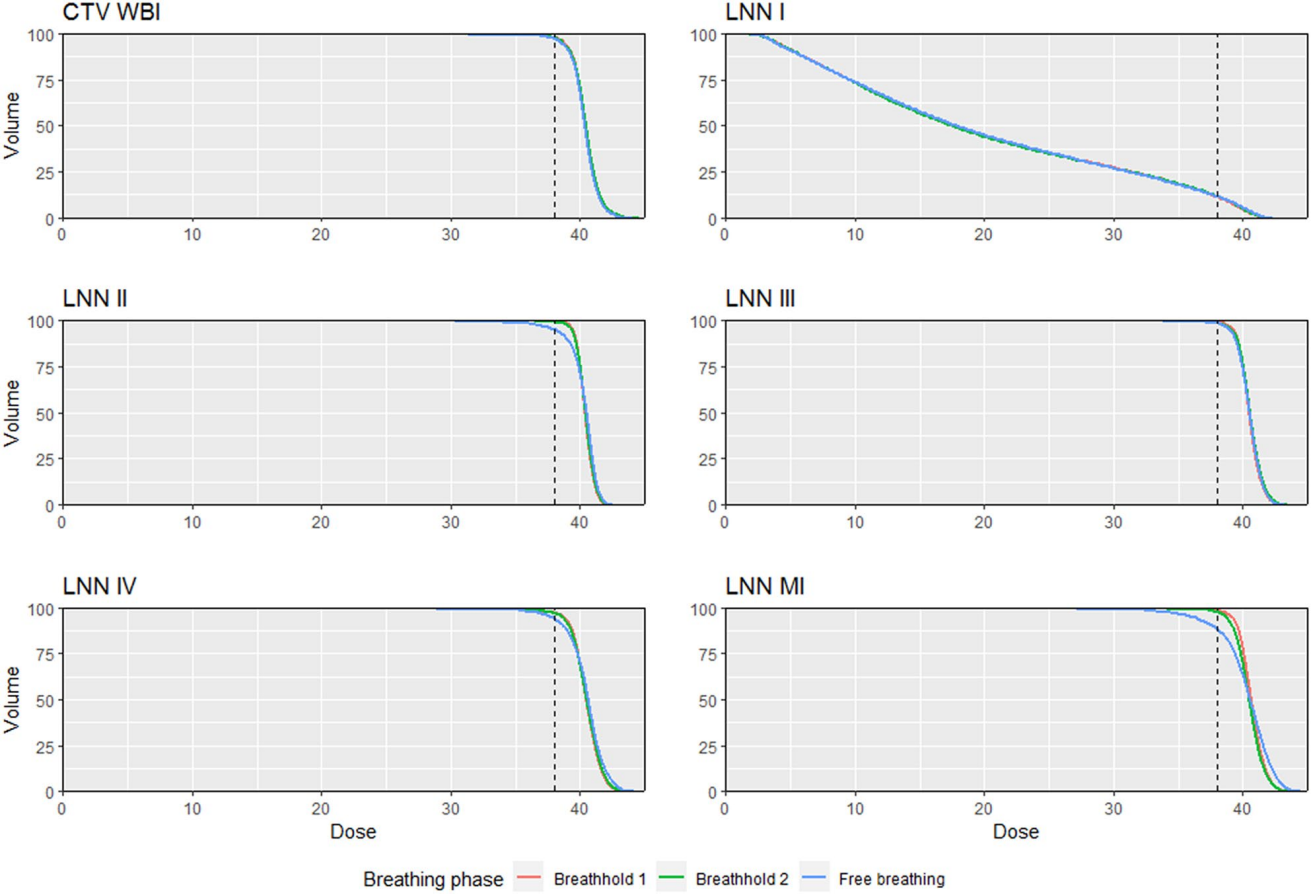
Dose-volume histogram showing mean (of patients) minimum dose received by given percentage of target volume. Almost all lines overlap, indicating the robustness of a photon radiotherapy treatment: only the internal mammary nodes (LNN MI) experienced higher dose inhomogeneity and underdosage in failure to breathhold anatomy. The vertical interrupted line represents 95% of the prescription dose.

**Figure 3:**
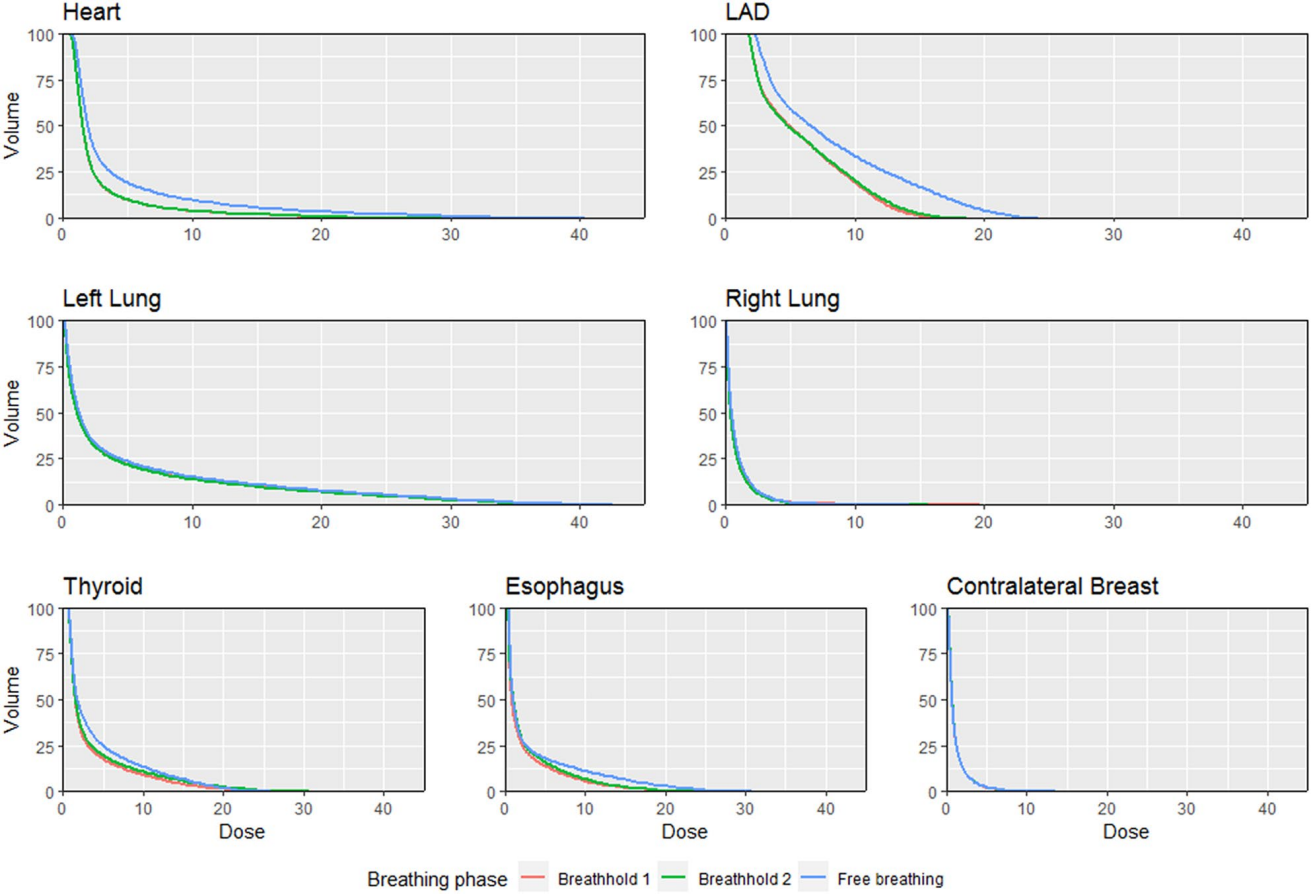
Dose-volume histogram showing mean (of patients) minimum dose received by given percentage of target volume. Contrary to TVs, failure to breathhold causes heart and LAD doses to increase, as compared to breathhold anatomy. Lines for repeated breathhold overlap, showing that although statistical differences exist, these are likely not relevant.

DVH parameters for TVs are reported in Table 2 and for OARs in Table 3. Figures 2 and 3 show mean DVH curves for TVs and OARs, respectively. The TVs showed no significant dosimetric differences for RBH. In FTBH, there were numerically significant but clinically less relevant dosimetric differences for CTV WBI, LNN III and LNN IV. Larger differences were apparent in D95 and D98 of LNN II and LNN MI, which were also significantly reduced in FTBH (Table 2). We report dose to the LNN I region, but did not include this region as a TV.

**Table 2:** Target volume dose parameters after transposition of breathhold plan (BH) to repeated breathhold (RBH) and failure to breathhold (FTBH) CT anatomy.

ROI	BH	FTBH			RBH		
	Dose (Gy) ± sd	Dose (Gy) ± sd	T-statistic	P-value	Dose (Gy) ± sd	T-statistic	P-value
**CTV WBI**							
Dmean	40.42 ± 0.17	40.33 ± 0.21	3.045	0.007	40.47 ± 0.18	− 0.508	0.617
D02	42.59 ± 0.71	42.44 ± 0.74	2.435	0.025	42.63 ± 0.75	− 1.496	0.151
D50	40.42 ± 0.13	40.37 ± 0.18	1.935	0.068	40.45 ± 0.16	− 1.461	0.160
D95	38.76 ± 0.44	38.55 ± 0.44	4.394	< 0.001	38.66 ± 0.49	2.293	0.033
D98	38.00 ± 0.66	37.69 ± 0.70	3.219	0.005	37.90 ± 0.75	1.679	0.110
**LNN II**							
Dmean	40.40 ± 0.44	40.26 ± 0.55	1.090	0.289	40.42 ± 0.36	− 0.210	0.836
D02	41.62 ± 0.85	41.81 ± 0.73	− 1.475	0.156	41.70 ± 0.84	− 1.096	0.287
D50	40.40 ± 0.43	40.51 ± 0.38	− 1.389	0.190	40.43 ± 0.38	− 0.644	0.527
D95	39.45 ± 0.33	38.06 ± 2.30	2.631	0.016	39.35 ± 0.39	0.846	0.408
D98	39.09 ± 0.48	36.57 ± 3.36	3.372	0.003	38.90 ± 0.61	1.120	0.277
**LNN III**							
Dmean	40.50 ± 0.53	40.49 ± 0.48	0.160	0.875	40.56 ± 0.65	− 1.251	0.226
D02	42.18 ± 0.98	42.24 ± 1.00	− 0.478	0.638	42.38 ± 1.11	− 2.963	0.008
D50	40.48 ± 0.52	40.53 ± 0.45	− 0.618	0.544	40.55 ± 0.61	− 1.543	0.139
D95	39.23 ± 0.45	38.98 ± 0.51	2.475	0.023	39.12 ± 0.86	1.056	0.304
D98	38.66 ± 0.93	38.34 ± 0.81	1.494	0.152	38.41 ± 1.55	1.393	0.180
**LNN IV**							
Dmean	40.44 ± 0.49	40.46 ± 0.96	− 0.079	0.938	40.46 ± 0.68	− 0.105	0.918
D02	42.48 ± 0.98	43.01 ± 1.23	− 2.401	0.027	42.64 ± 1.25	− 0.882	0.389
D50	40.48 ± 0.48	40.63 ± 0.69	− 1.223	0.236	40.51 ± 0.69	− 0.249	0.806
D95	38.67 ± 1.16	37.77 ± 2.50	1.581	0.130	38.60 ± 1.17	0.444	0.661
D98	37.51 ± 2.98	36.34 ± 3.50	1.365	0.188	37.54 ± 2.65	0.045	0.964
**LNN MI**							
Dmean	40.67 ± 0.36	40.23 ± 1.23	1.772	0.092	40.46 ± 0.76	1.370	0.187
D02	42.62 ± 0.82	43.37 ± 1.75	− 1.988	0.061	42.58 ± 1.05	0.271	0.789
D50	40.65 ± 0.38	40.55 ± 1.07	0.488	0.631	40.49 ± 0.76	1.144	0.267
D95	39.14 ± 0.22	36.09 ± 4.25	3.200	0.007	38.69 ± 1.12	1.859	0.079
D98	38.45 ± 0.47	33.90 ± 5.57	3.653	0.003	37.93 ± 1.63	1.528	0.143
**CTV LNN**							
Dmean	40.44 ± 0.33	40.36 ± 0.47	0.678	0.506	40.47 ± 0.42	− 0.525	0.605
D02	42.33 ± 0.91	42.65 ± 0.96	− 1.783	0.091	42.48 ± 1.04	− 1.599	0.126
D50	40.40 ± 0.27	40.50 ± 0.35	− 1.172	0.256	40.46 ± 0.35	− 1.010	0.325
D95	39.16 ± 0.26	38.03 ± 1.69	2.863	0.010	38.97 ± 0.58	1.503	0.149
D98	38.45 ± 0.93	36.44 ± 2.78	3.040	0.007	38.27 ± 1.09	0.954	0.352
**(LNN I)**							
Dmean	20.08 ± 4.95	20.14 ± 5.15	− 0.173	0.865	20.03 ± 4.98	0.910	0.374
D02	40.66 ± 0.75	40.92 ± 0.78	− 2.765	0.012	40.74 ± 0.81	− 0.743	0.467
D50	17.61 ± 7.93	17.80 ± 8.12	− 0.620	00.543	17.46 ± 7.93	2.518	0.021
D95	3.95 ± 4.02	3.82 ± 3.56	0.813	0.426	3.92 ± 3.94	1.008	0.326
D98	3.08 ± 3.07	3.06 ± 2.86	0.150	0.882	3.07 ± 2.98	0.190	0.852

CTV LNN is what we consider the unoperated axilla, i.e. LNN II + LNN III + LNN IV but excluding CTV MI (a target volume with separate planning optimization). (LNN I): this region is not included as a target volume in our trial, but we report dose to this region for the interested reader. Tests report comparisons of either FTBH or RBH to BH.

*CTV WBI* clinical target volume of the treated breast, *ROI* region of interest, *Dxx* minimum dose received by xx% of ROI volume, *LNN I through IV* lymph node levels I–IV, *LNN MI* internal mammary nodes, *Dmean* mean dose delivered to ROI volume.

**Table 3:** Organ at risk dose parameters after transposition of breathhold plan (BH) to second breathhold (RBH) and failure ot breathhold (FTBH) CT anatomy.

ROI	BH	FTBH			RBH		
	Dose (Gy) ± sd	Dose (Gy) ± sd	T-statistic	P-value	Dose (Gy) ± sd	T-statistic	P-value
**Heart**							
D02	14.09 ± 8.93	24.46 ± 7.16	− 7.453	< 0.001	14.21 ± 8.93	− 0.528	0.604
Dmean	2.47 ± 0.97	3.96 ± 1.35	− 7.509	< 0.001	2.50 ± 0.98	1.076	0.296
V5	7.79 ± 5.68	16.87 ± 7.68	− 6.909	< 0.001	7.97 ± 5.88	− 1.147	0.265
V10	3.62 ± 3.61	9.59 ± 5.59	− 6.787	< 0.001	3.76 ± 3.62	− 1.656	0.114
V20	1.36 ± 1.62	3.96 ± 2.82	− 6.074	< 0.001	1.39 ± 1.65	− 0.369	0.716
V30	0.45 ± 0.57	1.44 ± 1.25	− 4.594	< 0.001	0.45 ± 0.57	0.127	0.900
**LAD**							
D02	14.22 ± 10.16	21.22 ± 9.90	− 3.952	< 0.001	15.01 ± 9.80	− 1.323	0.201
Dmean	5.99 ± 4.19	8.33 ± 4.08	− 3.274	0.004	6.06 ± 3.94	− 0.514	0.613
V5	31.20 ± 25.57	48.45 ± 23.41	− 4.048	< 0.001	32.30 ± 24.50	− 0.922	0.368
V10	15.45 ± 20.14	27.50 ± 19.80	− 3.018	0.012	16.18 ± 19.69	− 0.861	0.400
V20	6.18 ± 14.28	10.03 ± 13.76	− 1.326	0.201	6.44 ± 13.22	− 0.527	0.604
V30	1.958 ± 5.30	2.52 ± 4.63	− 0.716	0.483	1.42 ± 4.20	− 1.784	0.090
**Left lung**							
D02	32.23 ± 2.03	33.36 ± 2.62	− 3.083	0.006	32.09 ± 2.42	0.665	0.514
Dmean	4.46 ± 0.68	4.83 ± 0.68	− 3.604	0.002	4.39 ± 0.71	1.538	0.141
V5	21.67 ± 3.09	22.99 ± 3.06	− 2.856	0.010	19.85 ± 5.45	2.266	0.035
V10	14.13 ± 2.59	15.36 ± 2.69	− 3.301	0.004	13.88 ± 2.68	1.331	0.199
V20	7.31 ± 1.69	7.93 ± 1.71	− 2.250	0.037	7.14 ± 1.63	1.277	0.217
V30	2.86 ± 0.87	3.37 ± 1.13	− 2.790	0.012	2.86 ± 0.97	0.082	0.936
**Right lung**							
D02	4.48 ± 3.12	4.44 ± 3.46	0.200	0.844	4.14 ± 3.04	2.351	0.030
Dmean	0.80 ± 0.54	0.87 ± 0.65	− 1.962	0.065	0.76 ± 0.55	2.022	0.057
V5	2.73 ± 4.44	3.06 ± 5.55	− 0.949	0.355	2.46 ± 4.40	2.712	0.014
V10	0.63 ± 0.83	0.61 ± 1.08	0.177	0.862	0.49 ± 0.81	1.837	0.082
V20	0.06 ± 0.18	0.01 ± 0.03	1.342	0.195	0.01 ± 0.03	1.194	0.247
V30	0.02 ± 0.07	0.00 ± 0.00	1.000	0.330	0.00 ± 0.00	1.000	0.330
**Thyroid**							
D02	18.91 ± 11.21	20.10 ± 14.87	− 0.701	0.492	21.53 ± 12.09	− 2.179	0.042
Dmean	3.38 ± 1.79	4.24 ± 3.25	− 1.708	0.104	3.76 ± 2.08	− 1.657	0.114
V5	14.37 ± 9.27	17.28 ± 15.80	− 1.298	0.210	16.18 ± 10.92	− 1.360	0.190
V10	7.72 ± 6.87	11.10 ± 11.90	− 1.793	0.089	9.69 ± 7.92	− 1.905	0.072
V20	3.39 ± 4.48	6.04 ± 7.76	− 1.967	0.064	4.55 ± 4.93	− 2.034	0.056
V30	1.27 ± 2.42	2.88 ± 4.69	− 1.782	0.091	1.79 ± 2.61	− 2.170	0.043
**Esophagus**							
D02	15.97 ± 4.64	22.37 ± 9.69	− 3.354	0.003	16.60 ± 7.72	− 0.547	0.591
Dmean	2.44 ± 0.77	3.41 ± 1.51	− 3.485	0.002	2.71 ± 1.57	− 1.179	0.253
V5	13.00 ± 7.00	16.14 ± 7.98	− 3.023	0.007	14.00 ± 10.97	− 0.767	0.452
V10	6.71 ± 4.33	10.54 ± 6.36	− 3.411	0.003	7.82 ± 7.49	− 1.111	0.281
V20	1.10 ± 0.97	3.92 ± 4.08	− 3.228	0.004	1.78 ± 2.59	− 1.365	0.188
V30	0.04 ± 0.08	1.36 ± 2.28	− 2.584	0.018	0.34 ± 1.11	− 1.216	0.239
**Contralateral breast**							
D02	5.28 ± 2.32	5.57 ± 2.62	− 1.449	0.164	5.47 ± 2.48	− 2.130	0.046
Dmean	1.11 ± 0.45	1.12 ± 0.48	− 0.675	0.508	1.12 ± 0.46	− 2.112	0.048
V5	3.17 ± 2.88	3.23 ± 3.21	− 0.328	0.746	3.23 ± 2.86	− 0.898	0.381
V10	0.034 ± 0.51	0.48 ± 0.67	− 2.446	0.024	0.42 ± 0.62	− 1.568	0.133
V20	0.01 ± 0.03	0.02 ± 0.08	− 1.231	0.233	0.01 ± 0.04	− 1.000	0.330

Tests report comparisons of either FTBH or RBH to BH.

*ROI* region of interest, *LAD* left anterior descending coronary artery, *sd* standard deviation, *Dxx* minimum dose received by xx% of ROI volume, *Vxx* volume percentage receiving at least xx Gy, *Dmean* mean dose delivered to ROI volume.

Regarding OARs, there were no relevant dosimetrical differences for RBH, with the exception of the V30 to the thyroid. In FTBH, there were significant dose differences for all OARs except for thyroid and right lung (Table 3).

## Discussion

This research evaluates the clinical impact of positional changes of RBH or FTBH in WBI + RNI including the LNN MI in prone position. We demonstrated that there is more positional variation for nodal TVs, LAD, esophagus and thyroid in RBH than previously observed for the breast itself^14^. The positional changes vary markedly more for all ROIs in FTBH. The impact of positional variation in RBH on dosimetry was limited, but the impact of positional variation in FTBH was more important.

There are several reasons why the prone position is not in widespread use in WB + RNI. Most treatment devices on the market support the patient’s shoulder while they are lying in prone position with extended arms. Irradiation of the lymph node regions in this position needs to go either through the supporting device and thereby creating a bolus effect that increases toxicity^11,27^, or using longer beam paths through healthy tissues, increasing OAR exposure and its associated risks. Furthermore, most prone positioning devices provide very little in the way of reproducible arm support^11^, which is especially relevant in WB + RNI because the target regions are influenced by arm position. Because of these limitations, most treating centers would have to invest in new devices in order to be able to perform optimal prone positioning. Furthermore, as observed in WBI^28,29^, longer treatment slots are likely to be necessary at least for some patients in the prone positioning. Despite the dosimetric benefits shown for decreased cardiopulmonary exposure with prone positioning in WB + RNI^9–12,30^, it is much easier to apply breathhold in the supine position to improve cardiopulmonary exposure using breathholding techniques that are already available at many centers^6,7^. For this reason, only a few papers report on WBI + RNI in prone position, almost always with free breathing^9–12,16,27,31^ and most do not treat LNN MI.

In a former publication from our group, Speleers et al.^12^ report on WBI + RNI including LNN MI in supine and prone position comparing photons with protons, but with free breathing. Comparing their free breathing prone position to the BH position in the current trial, mean doses for heart (4.3 vs 2.47 Gy), LAD (9.0 vs 5.99 Gy), ipsilateral lung (5.3 vs 4.46 Gy) and contralateral lung (0.91 vs 0.8 Gy) are in favour of breathhold. Our FTBH OAR doses are very similar to their free breathing treatment. However, proton treatment—either in supine or in prone position—is superior to our results for sparing OARs. Kainz et al. report WBI + RNI including LNN MI in prone position using helical tomotherapy, but using a different dose prescription of 45 Gy and a different prone positioning setup^10^. Their mean OAR doses for left sided breast cancer patients are all higher than the ones reported in our trial by a factor exceeding the ratio of prescribed doses (factor 1.12): heart (8.7 vs 2.47 Gy), ipsilateral lung (10.0 vs 4.46 Gy), contralateral lung (3.8 vs 0.8 Gy), esophagus (8.7 vs 2.44 Gy), thyroid (22.6 vs 3.38 Gy), contralateral breast (2.4 vs 1.11 Gy). The difference can be explained by a difference in prone set-up and the non-coplanar treatment possibilities of the prone crawl breast couch^19^ used in our trial.

This trial did not intend to evaluate the differences between breathhold or free breathing on OARs, but rather the consistency of intra-fraction RBH and dosimetrical impact of FTBH. Nevertheless, doses to OARs in FTBH anatomy are similar to those in a dedicated FB plan in prone position and better than in supine position^10,12^. The difference in overlap measures between breathhold and free breathing anatomy confirms prior observations that the heart changes position away from the treated fields^13^ and that breathhold increases lung volume decreasing the percentage of lung tissue being exposed to the treatment beams. Given these data, this trial indicates a probable benefit of breathhold when using prone positioning, and this is confirmed in data from our group^16^.

Comparing our results to reports from WB + RNI using breathhold in supine position, we see that compared to Yeung et al., our mean heart dose and LAD doses are higher (2.47 and 5.99 Gy vs 1.45 and 3.96 Gy, respectively). This puts their results in the same region of those observed with protons^12^. We believe this is mainly because of the higher importance we put on the LNN MI dose coverage as compared to their trial, where they aimed for the LNN MI to be covered by 80% of the prescription dose, whereas we demanded at least 95%. Despite this we still saw a lower volume of the ipsilateral lung receiving 20 Gy (V20) (16.3% vs 7.31%) In a retrospective study by Nissen et al.^32^, patients received 50 Gy and performed breathhold in supine position, but only 69% of left sided patients required WB + RNI and they did not cover LNN MI. Despite this, their mean heart dose for all left sided patients was still 2.69 Gy. This is very similar to our results, but keeping in mind we included LNN MI for all patients. They report an ipsilateral lung V20 of 24.85% for patients receiving nodal irradiation. Of all reports in this setting, Mohamad et al. is probably the most representative one to compare to our results. They included 22 left sided breast cancer patients requiring WB + RNI including LNN MI. Main differences are that they aimed to cover LNN MI with 90% of the prescription dose instead of our 95%, and the prescription dose seems to have been 50 Gy (although they don’t state their actual prescription dose and fractionation). They report mean heart and LAD doses of 2.23 and 9.40 Gy, and mean ipsilateral lung dose of 14.98 Gy and a V20 of 31.93%. Because of the dose and fractionation, any comparison should be made with caution. But cardiac doses at least seem similar using breathhold in prone position as compared to supine breathhold, but there is a clear benefit for reducing the ipsilateral lung dose using breathhold in the prone position.

Because treatment duration for each fraction is too long for each patient to undergo in a single breathhold without further training, our trial investigated whether the treatment is robust enough for RBH during a single treatment fraction. Our data demonstrate low variability in location and in dosimetric impact. The use of multiple RBH maneuvers within one treatment sessions increases treatment time. However, research on single prolonged breathholds of > 5 min in the prone position shows much promise in limiting these interruptions^33^.

The RBH technique used in this trial has the benefit of being very easily applicable. It has proven to be reproducible in WBI, with almost no movement of the treated breast^14^. We only use 2 Respisens magnetic sensors used to monitor if a breathhold is being performed and interrupt the beam manually if the patient fails to maintain breathhold. No automatic gating is required. This trial now shows that this technique can be extended to WBI + RNI including LNN MI, when a prone crawl breast couch is used.

Comparison with FTBH demonstrated the impact of patients not maintaining breathhold for the entire treatment, which is highly unlikely. As shown, dose distribution of the TVs is nearly identical, except for a slight underdosage to the LNN MI but without significant change in mean dose to this region. This could be of importance for the benefits that are observed in trials that include the LNN MI in the regional nodal targets^34–37^. These trials, however, used standard field setups for all patients. Borm et al.^38^ investigated the dose distributions that are achieved using these setups for some of the landmark trials in regional nodal irradiation. The mean dose to the LNN MI region in MA.20 and EORTC 10,981–22,023 was about 37.8 and 41.8 Gy, respectively, which corresponds to 76% and 84% of the prescribed dose of 50 Gy to this region. In our trial, the mean dose to the LNN MI region was not significantly different between RBH and FTBH, and the average minimum dose in FTBH is still 85% of the prescribed dose. The same can be said for region LNN II, that received a minimum dose of 91% of the prescription dose in our trial, still higher than the mean dose reported in this region for the MA.20 group that had more than 10 nodes removed or had less than 3 affected nodes, namely 88% of the prescription dose.

Furthermore, our minimal dose to the CTV LNN, which we consider the unoperated part of the axilla during axillary lymph node dissection (axillary regions II-III-IV), is still covered with a minimal dose of 91% of the prescribed dose in the case of FTB. In the current era of CT dose calculation with patient level optimisation and intensity modulation, this is certainly a suboptimal result. But this result is still superior to the classic field setups on which our current evidence for regional nodal irradiation is based. Unsurprisingly, we observed significantly higher OAR doses in FTBH, especially for heart, LAD, lungs and esophagus.

We did not include axillary region I into the TVs. This is the largest lymph node region, and including it might increase heart and lung doses, especially since this region is not covered “accidentally” in prone irradiation of the breast^39^. However, this region is surgically cleared in axillary lymph node dissection, whereas the other lymph node regions are (partly) avoided during standard axillary dissection. Therefore, our institution only includes level I in the case of positive sentinel node biopsy and no axillary dissection (AMAROS)^40^ or a high positive/total removed nodal ratio^41^.

Although the dose transposition method in itself proves that RBH can be used and FTBH leads to only small underdosages, we also report the overlap measures. Their importance is that they show that ROIs can have very dissimilar position between BH and FTBH (essentially free breathing) anatomy. The latter anatomy is often the one that is used for online CBCT or EPID matching prior to delivery of a treatment fraction. Matching on a different anatomy than the one used in treatment requires that the change from free breathing to breathhold position is consistently the same. In this trial, we only validated RBH, but not the validity of matching in free breathing and treating in breathhold. Therefore, we propose acquiring CBCT or EPID in breathhold and matching to the BH scan anatomy.

A point for improvement in this trial is that we delineated all regions of interest on CT images acquired without contrast while the RBH scan was a low dose CT, which is inherently more prone to artefacts. But because the RBH scan had no therapeutic implications, it was deemed more ethical to perform a low dose CT. Despite these issues, we found the delineation was not hampered for delineation of the TVs and major OARs, only the thyroid and esophagus were more difficult to identify on the RBH scan.

One caveat is that we only evaluated intra-fraction reproducibility of RBH, and not inter-fraction reproducibility. However, given the treatment robustness that we showed in this manuscript, it stands to reason that inter-fraction variability will have less impact than complete FTBH. Therefore inter-fraction variability will probably result in doses ranging between what we report for FTBH and RBH.

Our results shows that RBH can be performed using a simple technique and without fear for clinically relevant differences between the intended and delivered plan, provided that there is adequate image guided patient position verification at the start of each session. In the event of FTBH, TV coverage in photon radiotherapy will still be adequate, whereas OAR dose will increase.

## Conclusion

When using RBH, OAR and TV position in prone crawl position is reproducible for all the large ROIs while smaller volumes of interest, such as the LAD and the IMN show poorer overlap. All ROIs have similar dosimetry in RBH. For FTBH, TVs remain adequately covered but overlap is poor for most ROIs, and the heart, LAD, ipsilateral lung and esophagus receive higher doses. RBH shows the robustness required for clinical use, but CBCT should preferentially also be performed in BH.

## Data Availability

Data analysed during this study are available from the corresponding author on reasonable request within the confines of EU General Data Protection Regulations.
